# The Effect of Obesity and Pregnancy Weight Gain on Maternal and Child Health

**DOI:** 10.1155/2024/8816878

**Published:** 2024-11-20

**Authors:** Richard Gearhart, Nyakundi M. Michieka, Lyudmyla Sonchak-Ardan, Evan Stutzman

**Affiliations:** ^1^Department of Economics, 20 BDC, California State University, Bakersfield, 9001 Stockdale Highway, Bakersfield, California 93311, USA; ^2^Department of Economics, Susquehanna University, 514 University Ave., Selinsgrove 17870, Pennsylvania, USA; ^3^Susquehanna University, 514 University Ave., Selinsgrove 17870, Pennsylvania, USA

**Keywords:** BMI, fixed effects, infant health, maternal health, weight gain

## Abstract

In this study, we estimate the effect of prepregnancy obesity and excessive pregnancy weight gain on infant and maternal health outcomes. We rely on a large sample of maternally linked restricted data from 2004 to 2019 South Carolina birth certificates, which allow us to track the same mothers during multiple pregnancies over a period of more than 15 years. To address possible limitations of previous research, we account for genetic confounders and unobservable maternal and environmental factors by relying on a maternal fixed effects strategy. We find that gaining above recommended amounts of weight increases the likelihood of delivering a high weight infant by 2.34 percentage points, while being obese increases this likelihood by 2.58 percentage points. These large negative effects of weight gain outside recommended ranges, as well as the effects of being obese, are present in White and Black mothers. Also, our results indicate that mothers who gained too much weight, or were obese, had a higher likelihood of cesarean section and higher likelihood of being induced. Finally, among the subsample of Medicaid mothers, excessive pregnancy weight gain as well as inadequate weight gain increased the likelihood of NICU admission.

## 1. Introduction

In recent years, a growing fraction of women have been entering pregnancy outside a normal weight, making prepregnancy obesity one of the most common high-risk obstetric situations [1]. For instance, between 2011 and 2015, the prevalence of normal weight declined from 47.3 to 45.1% [2], and between 2016 and 2019, obesity rates increased for all maternal age groups [3]. Further, the rate of pregnancy complications linked to obesity, such as hypertensive disorders and caesarian sections, has been on the rise. Similarly, the share of women in the US with pregnancy weight gain (more technically referred to as gestational weight gain [GWG]) outside of recommended ranges has been going up. According to the Centers for Disease Control and Prevention (CDC), most pregnant women do not gain the recommended amount of weight. Recent studies indicate that only 32% of women gain the recommended amount of weight, with 48% gaining too much and 21% gaining too little [3]^1^. Given the importance of in-utero conditions and nutrition for fetal growth and development, and the fact that weight issues are potentially modifiable risk factors, it is important to assess the effects of maternal obesity and excessive pregnancy weight gain [4]. In this study, using restricted and unique panel birth certificates data from 2004 to 2019 from South Carolina that encompass the universe of all multiparous births, we examine whether, after accounting for genetic, maternal, and environmental confounders, maternal obesity and excessive GWG have any effect on infant outcomes and maternal health at delivery.

GWG and prepregnancy obesity (as measured by body mass index [BMI] greater or equal to 30), are critical factors affecting both infant and maternal health. A large body of literature examines the association between GWG and/or BMI and a variety of child outcomes. The majority of previous studies rely on multivariate regressions, in some cases with limited controls to account for confounders, flawed design, and other methodological issues [5]. Overall, these studies suggest a positive association between excessive GWG/obesity and infant birth weight, the likelihood of macrosomia, and delivering a large-for-gestational-age (LGA) infant [6]. Alternatively, gaining too little weight, or being underweight, is associated with delivering a lower weight infant, as well as a small-for-gestational-age (SGA) infant [7, 8]. There is suggestive evidence that excessive GWG is associated with childhood obesity [9, 10], a lower Apgar score [11], and childhood asthma [5]. While the research on GWG/BMI and maternal outcomes is limited, studies find that excessive GWG and higher BMI are associated with a higher likelihood of cesarean section, hypertension, preeclampsia, postpartum hemorrhage [8, 12, 13], and a lower likelihood of breastfeeding initiation [14–16].

The major limitation of the existing studies is that the association between GWG/prepregnancy BMI and various outcomes is not causal and can be attributed to other underlying factors that impact GWG/obesity and outcomes of interest [5]. For instance, genetic factors shared by a mother and a child could impact maternal weight gain and simultaneously influence infant outcomes [17]. Alternatively, unobservable health endowments and health, and nutrition attitudes could also affect maternal GWG/obesity and child outcomes. Without being able to control for, or measure, these confounders, we might be mixing the effects of these confounders with the effects of GWG/obesity. To our knowledge, only Ludwig and Currie [17], and Yan [18], account for unobservable confounders by relying on a maternal fixed effects strategy. Ludwig and Currie [17] focus on data from Michigan and New Jersey and find that women who gain more than 25 kg during pregnancy are more likely to deliver heavier infants compared to those who gain 8–10 kg. One major limitation of their study is the absence of control for maternal BMI. Yan [18], using data from Pennsylvania and Washington, finds that excessive GWG and obesity increase the risk of delivering a high birth weight baby, while gaining too little weight and being underweight increase the risk of low birth weight.

To account for unobservable genetic, maternal, and environmental confounders, we follow Ludwig and Currie [17] and Yan [18] by relying on a maternal fixed effects strategy. We expand and build on previous works in several ways. First, we explore whether the amount of weight gain and obesity impacts maternal health at delivery, including the likelihood of precipitous labor, labor induction, augmentation, cesarean section, and breastfeeding. These outcomes have not been previously explored in the context of fixed effect estimation. Second, we reexamine the effect of GWG and obesity on a range of infant outcomes using more recent data that encompasses all sibling births in the state of South Carolina over a period of 16 years. Third, our large sample size of unique restricted data allows us to obtain precise estimates and explore the heterogeneity of the effect by race. While there are large racial disparities in the obesity and weight gain patterns, analysis by race has been largely omitted in the existing literature due to small sample sizes.

Our results indicate that obesity and excessive GWG increase the likelihood of delivering a high birth weight infant by 35.83% and 32.5%, respectively. Similarly, gaining too little weight or being underweight increases the likelihood of delivering a low birth weight infant by 30% and 38.27%, respectively. These effects are similar in magnitude for White and Black mothers. We also find that obesity and high GWG increase the likelihood of cesarean section and labor induction for White and Black mothers. Also, excessive GWG increases the likelihood of labor augmentation for White mothers, while inadequate weight gain has a small negative effect on the likelihood of breastfeeding initiation among Black mothers. Given that weight gain during pregnancy, as well as maternal BMI, is potentially modifiable through various individual and public health interventions, our results highlight the importance of increased focus on maternal weight issues.

## 2. The Economic Burden of Negative Maternal and Infant Health Outcomes

The link between obesity and excessive GWG (EGWG) on negative health outcomes has been studied extensively over time. Guelinckx et al. [19] note that risk for a caesarean-section (C-section) delivery is more than doubled from EGWG. Given that C-section recovery times are traditionally longer than vaginal birth recovery times, this provides evidence that unhealthy weight gain has significant and long-lasting impacts. Maternal obesity is one of the biggest risk indicators during pregnancy [2]. For instance, Guelinckx et al. [19] note that maternal obesity is associated with a higher risk of polycystic ovary syndrome, higher rates of miscarriage, gestational diabetes, preeclampsia, labor induction, and C-sections and doubles the risk of death for stillbirth and perinatal deaths.

Even more critical is the link between EGWG and/or maternal obesity on childhood health outcomes. Khalak, Cummings, and Dexter [20] note that infants of obese mothers are more likely to be delivered by C-section, have larger birth weights, and require assisted ventilation in the NICU admission. Ren et al. [21] note that high birth weight babies have a higher probability of being considered overweight or obese as a child and adolescent. According to Kapral et al. [22], children who are born with a high birth weight, which is linked to EGWG and maternal obesity, have a higher probability of becoming overweight or obese later in life. According to the University of California San Francisco (UCSF), parental obesity is also a significant predictor of childhood obesity; if one (two) parent(s) are obese, the probability of a child being obese is 50 (80) percent. Given that both obesity and EGWG have significant behavioral components, including diet and exercise, the likelihood of considerable cost externalities exists [23, 24].

The economic consequences of obesity are well understood. Cawley and Meyerhoefer [25] find that the traditional estimates for obesity are too low; in 2005 dollars, they estimate that being obese is associated with an additional annual cost of over $2700^3^. Obesity increases the likelihood of needing a C-section, where C-sections are traditionally more expensive than vaginal deliveries. In fact, even with employer-sponsored health insurance, the average cost of a C-section to the mother is nearly $1000 higher [26]. In addition, given the link between maternal obesity and other negative infant health outcomes, including NICU admission, Azher et al. [27] found that maternal obesity is associated with both prolonged maternal and infant stays, increasing costs^4^. Given the relatively expensive nature of NICU treatment, any increase in the likelihood of this is likely to create significant healthcare cost burdens. In fact, Bhattacharya and Bundorf [28] attribute some of the gender discrimination wage gap due to female obesity; they observe that obese women get lower wages, in part due to higher health insurance premiums, based on the probability of negative pregnancy outcomes.

## 3. Data

To estimate the impact of obesity and GWG on infant and maternal outcomes, we rely on data between 2004 and 2019 from South Carolina birth certificates. We use a restricted version of the data provided by the South Carolina Department of Health and Environmental Control (DHEC), which links births to the same mother across time, allowing us to track mothers over the course of more than 15 years, if they have given birth multiple times. The data include detailed information on infant health at birth, basic demographic characteristics of the mother, prenatal care information, prepregnancy and pregnancy risk factors, characteristics of a delivery and onset of labor, and information on previous pregnancy. In 2003, South Carolina was one of the first states that implemented revised birth certificates. The revisions incorporated new data not previously available, including data on prepregnancy BMI, breastfeeding initiation, WIC participation, and payment methods.

Using data on prepregnancy BMI, we identify women who are underweight (BMI < 18.5), normal weight (BMI > 18.5 and BMI < 24.9), overweight (BMI ≥ 25 and BMI < 29.9), and obese (BMI ≥ 30). According to the 2009 Institute of Medicine (IOM) guidelines, women who are underweight should gain 28 to 40 pounds during pregnancy; those with normal weight should gain 25 to 35 pounds; overweight women should gain 15 to 25 pounds and those who are obese should gain 11 to 20 pounds [29]. We create a dummy variable for each weight gain category, indicating whether a mother gained an excessive amount of weight, an inadequate amount of weight, or the recommended amount of weight for a given BMI, as well as categorize mothers into those who are obese, overweight, normal weight, and underweight. We follow Ludwig and Currie [17] and restrict our sample to singleton births that are full-term (≥ 37 weeks and < 41 weeks of gestation), as GWG guidelines are provided for full-term births. Furthermore, we drop observations with unusually high infant birth weight (> 7000 g), or unusually low weight (< 400 g). Also, since our estimation strategy is based on maternal fixed effects, we drop mothers who had only one birth during our sample period, as these singleton observations do not aid in our identification.

As our outcome measures, we focus on a continuous measure of infant birth weight, the likelihood of being born low birth weight (< 2500 g), the likelihood of being born high birth weight (> 4000 g), and the likelihood of NICU admission. Infant weight has been considered an important marker of health at birth that could cause immediate and long-term health consequences [30–32]. We also focus on maternal characteristics of delivery, including the likelihood of having a precipitous labor, labor augmentation, labor induction, and C-section, which are some of the complications during delivery that can pose risks to a mother and a child [33–35]. Finally, we look at the likelihood of breastfeeding.

Table 1 provides summary statistics for our overall sample, and subsamples stratified by race and Medicaid status.

For comparison, in the first column, we show our multiparous sample of all full-term births in South Carolina. In the second and third column, we present our multiparous births sample stratified by race. In the fourth column, we show summary statistics for mothers on Medicaid, and in the remaining two columns, we focus on Medicaid mothers by race. In our full sample, approximately 29% of mothers were obese, with 45% gaining too much weight. Examining the sample by race indicates that Black mothers are more likely to have inadequate weight gain and less likely to gain excessive amounts of weight compared to White mothers; however, Black mothers are less likely to be underweight and much more likely to be obese in comparison to their White counterparts. Black mothers are also more likely to have lighter infants and low birth weight infants, are less likely to have high birth weight infants, and are less likely to breastfeed relative to White mothers. A comparison of demographic characteristics indicates that Black mothers are more likely to be younger and have prepregnancy diabetes, hypertension, and previous cesarean sections. However, Black mothers are less likely to smoke before and during pregnancy compared to White mothers.

### 3.1. Empirical Model

To estimate the effects of obesity and GWG on infant and maternal outcomes, we rely on the following empirical specification:

(1) $$\begin{array}{c} Y_{ijtc}=\beta_{1}{\text{BMI}}_{ijtc}+\beta_{2}{\text{GWG}}_{ijtc}+\beta_{3}X_{ijtc}+\mu_{j}+\gamma_{t}+\lambda_{c}+\varepsilon_{ijtc}, \end{array}$$

where for child *i,* born to mother *j* at time period *t* in county *c*, *Y* is a measure of child health at birth or maternal outcomes, BMI is maternal prepregnancy BMI category including obese, overweight, and underweight (normal BMI is an omitted category), GWG is a categorical variable for a GWG being excessive or inadequate (normal GWG is an omitted category), *X* is a vector of observable maternal and child characteristics such as a child's gender, maternal age dummies, education dummies, race, whether prenatal care originated in the first trimester, length of gestation, prepregnancy and pregnancy smoking, previous C-section, and whether a mother participated in the Women, Infants, and Children Supplemental Nutrition Program (WIC). We also include *γ* and *λ* for year and county fixed effects. Finally, μ is unobserved genetic characteristics or maternal health behaviors, and *ε* is an error term.

Using ordinary least squares to estimate the above equation can provide biased estimates, as some unobservable genetic, maternal, and environmental characteristics can impact both the likelihood of being obese and weight gain, as well as our outcomes of interest. For instance, excessive GWG and/or obesity can be related to the high birth weight of an infant, since a mother and an infant share the same genetic material. If we ignore these genetic factors, we may be attributing high birth weight to high gestational weight/obesity, ignoring the role of genes [17]. Alternatively, some unobservable maternal characteristics such as maternal health attitudes could impact her weight and infant health at birth. For example, health-conscious mothers will be more likely to watch their weight. The same mothers would also deliver healthier babies, regardless of weight gain/obesity. To account for these types of confounders, we rely on a maternal fixed effects estimation strategy. By focusing on the sample of mothers with multiple births, we can compare birth outcomes of the same mothers as they change their weight gain and BMI categories across births, differencing out the unobserved component μ in our empirical specification. Alternatively, we can think about the fixed effects strategy as regressing demeaned outcomes on demeaned dependent variables which, again, allow us to eliminate the unobserved component μ. Hence, this within-mother estimation strategy enables us to eliminate genetic confounders, environmental, and personal characteristics that are constant across pregnancies.

While a fixed effects strategy is commonly implemented to control for time-invariant confounders, including in some of the most recent health economics studies [36, 37], there are some limitations inherent within this technique. First, we are assuming that genetic and unobserved maternal characteristics are time-invariant. While this may be true about genetic factors, it is possible that some of the unobserved factors could vary across pregnancies. For instance, some mothers may change their health and nutrition habits in response to previous pregnancies. To mitigate these potential issues, we account for a wide set of prepregnancy and pregnancy-related factors. As a robustness check, we also restrict our sample to only Medicaid mothers, which allows us to account for family income changes over time.

Our fixed effects strategy may bias the results towards zero in the presence of a random measurement error [38]. Since weight gain and BMI information are reported by a woman after delivery, it is possible that some mothers may mistakenly recall this information leading to measurement error. However, according to a validation study, weight gain reported in birth certificates coincides with medical records 82.8% of the time [17, 39].

## 4. Results

Table 2 presents our maternal fixed effects estimates of the effect of GWG and prepregnancy BMI on infant outcomes for the entire sample of births in the state of South Carolina. While we primarily focus on the effects of maternal obesity and excessive weight gain, we also report estimates for other weight categories. Our estimates are also broken down by major racial groups.

Importantly, in both our full and racially stratified samples, we consistently find evidence that prepregnancy obesity and excessive GWG impact birth weight. For instance, in our full sample, being obese or having excessive GWG leads to birth weights that are 74.8 and 58 g higher than being normal BMI or gaining an adequate amount of weight. We also see that having a less than ideal BMI (underweight and overweight) as well as gaining less than recommended amount of weight leads to changes in infant birth weight. For instance, being underweight (overweight) leads to a birth weight that is 29.6 (23.6) grams lower (higher) than individuals with a normal BMI. Regardless of whether we stratify the sample by looking at White mothers or Black mothers, our results still hold.

Continuous measures of birth weight, however, may mask important effects within the birth weight distribution. Hence, we further examine the impacts of BMI and GWG on the likelihood of being born high birth weight or low birth weight. Being obese or overweight before pregnancy significantly increases the risk of delivering high birth weight infants, by 2.58% and 0.69% points (pp), respectively. Interestingly, we see that the impact of obesity on high birth weight infants is higher for White mothers than for Black mothers; being obese increases the likelihood of a high birth weight baby by 3.56 pp for White mothers, while increasing the likelihood by 1.3 pp for Black mothers. Similarly, mothers who have excessive GWG are 2.34 pp (32.5%) more likely to deliver a high birth weight baby, relative to mothers whose weight gain is within the normal range. Given the quite significant health costs imposed by obesity, we see that the impact of excessive GWG, being overweight, and being obese has large negative effects on infant outcomes, potentially increasing obesity rates in the future and further burdening the healthcare delivery system with additional costs.

On the other hand, we find that gaining inadequate GWG increases the likelihood of an infant being born low birth weight by 0.87 pp (30%). Being underweight has a larger percentage point impact on the likelihood of low birth weight than an inadequate GWG, with a 1.11 pp increase (38.27%). The impact of inadequate GWG is similar across races; however, being underweight only has an effect on the likelihood of delivering a low weight infant for Black mothers: this increases the likelihood of a low birth weight infant by 2.66 pp. Given that the prevalence of low birth weight is higher for Black mothers, these point estimates translate into a 56.60-percent increase for Black mothers when evaluated at the mean.

Next, we investigate the impact of suboptimal -maternal weight on NICU admissions. For our full sample, we see that only inadequate GWG is associated with a higher likelihood of NICU admission, of 0.17 pp. We see that these results are solely driven by White mothers, who have a 0.29 pp increase in the likelihood of NICU admission from inadequate GWG. One explanation may be monetary; Black mothers are less likely to have private insurance and more likely to have public insurance, which may impact the feasibility of utilizing NICU care.

Table 3 presents maternal fixed effects estimates of the effect of obesity and EGWG on maternal outcomes. First, we examine breastfeeding initiation. According to Weimer [40], the economic benefits of breastfeeding are substantial and indisputable. Cohen et al. [41] note that factors associated with breastfeeding initiation include being a nonsmoker, having a vaginal (rather than cesarean) delivery, and mother's education level. Interestingly, we find no adverse impact of excessive GWG, being obese, or being overweight on breastfeeding initiation, suggesting that the other risk factors found in Cohen et al. [41] are more impactful. However, we find evidence that inadequate GWG reduces the likelihood of breastfeeding initiation by 0.58 pp in our full sample and by 0.49 pp in our sample of White mothers.

Precipitous labor, which is defined as labor that happens quickly, is impacted by inadequate GWG and being obese, where having inadequate GWG (being obese) increases (decreases) the likelihood of precipitous labor by 0.38 (0.49) pp. Labor augmentation, which is defined as stimulating the uterus to increase the frequency, duration, and intensity of contractions after the onset of spontaneous labor, is mainly linked to GWG. We see that excessive (inadequate) GWG leads to a 0.46 (0.43) pp increase (decrease) in the likelihood of labor augmentation.

Of more practical importance are the impact of suboptimal maternal weights on labor induction and the type of delivery (vaginal versus cesarean). According to the Mayo Clinic, the primary reason for inducing labor is when there is concern for the mother's or baby's health^5^. We find that being obese and gaining excessive amounts of weight increase the likelihood of labor induction by 2.45 and 1.47 pp, respectively. Obesity has a very high likelihood on labor induction especially among Black mothers, equivalent to 3.79 pp, or 128% increase when evaluated at the mean.

As described earlier, cesarean sections are costlier than vaginal deliveries [42]. Witt et al. [42] also find that prior C-sections are a strong predictor of receiving a C-section for future births, as well as imposing higher morbidity and mortality rates for women and children. They also note that C-sections are associated with higher rates of postpartum care. Our results in Table 3 indicate that being obese, being overweight, and excessive GWG significantly increase the likelihood of C-sections. Being obese (overweight) leads to a 1.92 (0.52) pp increase in the likelihood of a C-section, which would increase the frequency of C-sections by 6.44 (1.74) percent when evaluated at the mean. We see that excessive GWG leads to a 0.53 pp increase in the likelihood of a C-section, which would increase the frequency of C-sections by nearly 2% at the mean. When looking at outcomes by race of the mother, we see that our results for the impact of excessive GWG on the likelihood of having a C-section are driven by Black mothers, who are nearly 2 times more likely to have a C-section from excessive GWG than White mothers. Conversely, the impact of being obese or overweight on the likelihood of a C-section is driven by White mothers, where obesity increases the likelihood of a C-section by 2.17 pp (7.28%) for White mothers.

Importantly, Tables 2 and 3 control for two potential indicators of differential outcomes for infants and mothers: previous C-section, and whether this is the first birth for the mother. Witt et al. [42] note that prior C-sections influence future C-sections, with concomitant potential negative birth outcomes for mothers and infants; however, more evidence suggests that this may not be medically necessary. Importantly, given the fact that we still see significant impacts of GWG and prepregnancy, BMI on infant and maternal health outcomes suggests that our results may be considered causal.

A number of papers have found a link between pregnancy payment sources and the probability of having a C-section, as well as the link between financial considerations and C-section rates [43–45]. Therefore, given that Medicaid pays providers different reimbursement rates at the state level and given that states often adopt Medicaid policies that are aimed at decreasing costs by lowering procedure use, we perform the same analysis, but on mothers in South Carolina who are on Medicaid. Infant results are presented in Table 4, while maternal results are presented in Table 5. In Table 4, we see similar results to our full sample; excessive or inadequate GWG leads to suboptimal birth weights, while uniformly increasing the probability of NICU admission for the overall Medicaid sample. In fact, excessive or inadequate GWG leads to 3.3 and 1.6 times the likelihood of NICU admission for mothers on Medicaid, compared to all mothers. We also see that being obese or overweight before prepregnancy (just like for the full sample) leads to no change in the likelihood of NICU admission. The results for the subsamples of Medicaid mothers stratified by race are similar to the overall samples of Black and White mothers.

In Table 5, we again see that results are qualitatively similar for our subsample of Medicaid mothers (including stratification by race), relative to all mothers, for maternal outcomes. This would suggest that though Medicaid may attempt to limit certain procedures, these may be more standardized for pregnancy, and so are less likely to be cut down on in cost-restraining attempts. In fact, it appears that Medicaid mothers are more likely to have induced labor than all mothers based on GWG and prepregnancy BMI, with Medicaid mothers who are obese (overweight) being 3.52 (2.13) pp more likely to have an induced labor than all mothers. It appears that Medicaid payment incentives may play a role in this, as Medicaid mothers who are overweight, unlike all mothers in our sample, have no difference in the likelihood of a C-section. While Medicaid mothers who are obese are 0.99 pp more likely to have C-sections, which is half the rate for all mothers in our sample.

The mechanisms between excessive weight gain/prepregnancy BMI and adverse maternal and infant outcomes are not well understood [46]. However, two potential independent pregnancy risk factors that could lead to greater pregnancy complications in response to higher weight gain or maternal obesity could be the development of gestational diabetes and pregnancy hypertension. Our supplementary analysis using birth certificates data suggests that being obese/overweight increases the likelihood of gestational diabetes. Similarly, being obese, overweight, and gaining excessive gestational weight lead to a higher likelihood of gestational hypertension. We do not include these results in the main text, as we do not know the sequence of these risk factors. For instance, mothers who are obese are more likely to develop gestational diabetes, but those mothers who have gestational diabetes are more likely to gain more weight during pregnancy. In this context, it is difficult to make a causal inference. When it comes to adverse infant outcomes, research suggests that maternal prepregnancy BMI and GWG may partly impact cardiometabolic health of the offspring through direct intrauterine mechanisms [47].

### 4.1. Cost Burden Estimates

Importantly, prepregnancy obesity rates have been increasing steadily in the United States. According to Driscoll and Gregory [48], prepregnancy obesity rates have increased by nearly 3% points between 2016 and 2019. Using our estimates, along with assuming that overweight and EGWG rates have increased in a similar manner, we estimate the total per-all pregnancy cost and total per-all Medicaid pregnancy cost increases from these trends. We do this using our estimates for all mothers in Tables 2 and 3 and for Medicaid mothers in Tables 4 and 5.

The average cost of a NICU stay is $76,000. We utilize MEPS data to assess the impacts of pregnancy complications on birth costs; the average cost of pregnancy complications added $5667 per person, which is likely an underestimate for the additional cost of C-sections^6^. We are then able to calculate the per-birth and per-Medicaid patient cost increases in South Carolina from several major outcomes, based on maternal EGWG, maternal overweight, and maternal obesity levels: (i) EGWG, maternal overweight, and maternal obesity levels leading to increased chances of C-sections, and (ii) EGWG, maternal overweight, and maternal obesity levels leading to increased NICU admission. Note that we do not include the costs of future obesity in our estimates nor do we include other additional economic costs (lost productivity, education outcomes, etc.).

For all births in South Carolina, the impact of a 3-percent increase in the fraction of pregnant women who suffer from EGWG, maternal overweight, and maternal obesity (individually) on NICU admission, and the likelihood of a C-section, increases per-birth total costs by $1087.71 (in 2019 dollars) on all mothers who gave birth. If we restrict our cost increases to only those mothers impacted, this increases their per-birth total costs by nearly $11,000 per birth. This suggests considerable external effects from EGWG, overweight, and obesity gains during pregnancy. When we restrict our sample to Medicaid mothers only, the numbers are even bigger. Relative to all mothers who gave birth on Medicaid, the impact of a 3-percent increase in the fraction of women who suffer from EGWG, maternal overweight, and maternal obesity (individually) on NICU admission, and the likelihood of a C-section, increases per-birth total costs by $2524.81 (in 2019 dollars) on all mothers who gave birth. Restricting the sample to only those mothers who saw these EGWG, overweight, and obesity changes, we would see an increase in per-birth total costs of $41,677.86. This highlights the considerable external burden again that weight gain and overweight/obesity rates have on healthcare costs related to pregnancy.

In fact, this three-percentage point increase in EGWG, overweight, and obesity rates among pregnant Medicaid women would affect less than one percent of all Medicaid recipients but increase total South Carolina Medicaid spending by nearly nine percent, highlighting how costly these conditions can be, especially given their relationships to NICU admission and C-section rates.

## 5. Discussion and Conclusion

In this study, we estimate the impact of prepregnancy obesity and excessive pregnancy weight gain on infant health at birth, as well as maternal characteristics of delivery, by relying on a sample of 2004–2019 full-term births among South Carolina mothers. The unique panel nature of our data allows us to track the same mothers over more than 15 years. By implementing a fixed effects strategy and comparing outcomes to the same mothers as they change their weight gain across pregnancies, we are able to account for unobserved genetic, individual maternal, and environmental confounders. Also, the large dataset allows us to examine the heterogeneity of estimated effects by race, an analysis that has not been performed in previous studies.

Our results indicate a deleterious effect of prepregnancy BMI outside of normal ranges and poor GWG for infant and maternal health outcomes. The magnitudes of the estimated effect are particularly large at the bottom and the top of birth weight distribution. Gaining too much weight, as well as being overweight and obese, increases the risk of delivering a high weight infant by 32.5%, 9.58%, and 35.83%, respectively. Gaining too little weight, or being underweight, increases the likelihood of low birth weight by 30% and 38.27%, respectively. While the patterns of GWG and prepregnancy obesity differ by race, the deleterious consequence of our major variables of interest is similar in signs and magnitudes across racial groups. The magnitudes of the effects in our study are larger in comparison to Yan [18]. For instance, we find that being obese increases the probability of high birth weight by around 35.83%, while Yan's [18] estimate is around a 26% increase. These discrepancies could be explained by the different sample composition, as we focus on the sample of South Carolina births, and there may be a potential heterogeneity of estimated effects across various geographic areas.

When looking at maternal outcomes, we do not find a negative effect of prepregnancy BMI or EGWG on breastfeeding. We do find that inadequate GWG reduces breastfeeding initiation, but only among Black mothers, and by only 2%. Excessive GWG increases the likelihood of labor augmentation and inducement among White mothers. Being obese increases the likelihood of labor inducement by around 57% for White mothers and 128% for Black mothers. Finally, both higher GWG and obesity and overweight status increase the likelihood of a C-section, with obesity having the largest impact of around 5–7 percent, depending on the sample of mothers. In the subsample of Medicaid mothers, we find that excessive weight gain and inadequate weight gain both increase the likelihood of NICU admission by around 17% and 12%, respectively.

Our results point out the benefits of potential interventions aimed at managing gestational weight and maternal BMI. Using conservative estimates, interventions that reduce EGWG, overweight, and obesity increases during pregnancy could save nearly $1100 per birth in healthcare expenses for all mothers giving birth; focusing only on the mothers who suffer these weight gains, interventions could reduce prebirth total costs by nearly $11,000.

Given the deleterious effects of excessive weight gain during pregnancy, clinicians and practitioners in the field should be more mindful of weight gain issues and recommend lifestyle interventions to manage weight during reproductive years. For instance, one of the potential inventions that has been previously implemented with some success is remote mobile application weight monitoring which provides real-time data to obstetricians and allows them to implement potential interventions [49]. Cantor [50] found that counseling and active behavioral interventions could help limit GWG. Dietary counseling also appears to have led to a beneficial, yet modest, improvement in GWG [51]. Finally, we need to acknowledge and overcome obstacles that some women and their obstetricians may face, including a lack of referrals for gestational weight management, lack of community resources, and patient inability to pay for specific resources/interventions [52].

One of the limitations of our study, which is inherent within the fixed effects literature, is the focus on mothers with multiparous births, and our results may not generalize to mothers who only have had one birth. Also, our analysis is restricted to Medicaid births in South Carolina, a state relatively more disadvantaged: South Carolina ranks 38th, based on various health indicators^7^ relative to healthier states such as Washington (rank 7) and Pennsylvania (rank 23) examined in Yan [18]. Hence, we believe that our results would be more generalizable to states demographically similar to South Carolina. Since we utilize the IOM weight gain guidelines for full-term pregnancies, we focus on full-term infant births only, and we are not able to assess the impact of obesity and excessive weight gain on preterm birth outcomes, which could be related to obesity and weight gain during pregnancy. As we point out in our empirical section, while a measurement error in reporting weight gain remains a potential issue, it is of less concern given the results in data validation studies [17]. Also, according to a recent study that specifically compares the South Carolina Birth Certificates data with electronic medical records to evaluate the accuracy of GWG and BMI records, there are high levels of correlation between these two datasets, indicating that self-reported measurement errors are unlikely to bias our results [53].

Overall, we find large negative effects of obesity and excessive weight gain during pregnancy on infant health outcomes. The effects on maternal outcomes are smaller, yet still sizable in the case of labor inducement and the likelihood of a C-section. In most cases, being obese prior to pregnancy has the largest negative effects. While changes in prepregnancy BMI may be more difficult to alter from a policy standpoint, changes in gestational weight could be potentially modifiable during the prenatal period through public health intervention policies, including raising awareness about the role of GWG, offering nutrition education and counseling, community-based strategies, incentivizing healthy levels of physical activity, and other interventions aimed at supporting healthy infant and maternal outcomes. Our results also point to the role of health care professionals, including obstetricians, midwives, and dietitians when it comes to assisting women with successful weight management during pregnancy. Ultimately, a stronger focus on GWG and prepregnancy BMI, both modifiable factors, could potentially confer large benefits to infant and maternal health.

## Figures and Tables

**Table 1:** Sample summary statistics.

Variables	All mothers	Black mothers	White mothers	Medicaid mothers	Medicaid Black	Medicaid White
Birth weight	3334.680 (454.611)	3185.504 (428.642)	3412.580 (448.645)	3265.598 (448.520)	3169.303 (424.632)	3353.863 (451.901)
Low birth weight	0.029	0.047	0.019	0.037	0.342	0.258
High birth weight	0.072	0.033	0.092	0.053	0.405	0.461
NICU	0.023	0.023	0.023	0.023	0.022	0.024
Breastfeeding	0.664	0.488	0.750	0.536	0.443	0.619
Precipitous labor	0.043	0.046	0.041	0.044	0.047	0.042
Labor augmentation	0.227	0.236	0.221	0.228	0.235	0.220
Induced labor	0.312	0.296	0.322	0.310	0.293	0.326
Cesarean section	0.298	0.305	0.295	0.288	0.296	0.281
Inadequate weight gain	0.264	0.331	0.228	0.299	0.342	0.258
Excessive weight gain	0.449	0.413	0.470	0.433	0.405	0.461
BMI: underweight	0.041	0.033	0.044	0.047	0.035	0.057
BMI: overweight	0.251	0.256	0.249	0.250	0.251	0.249
BMI: obese	0.290	0.399	0.237	0.333	0.395	0.279
Child male	0.509	0.506	0.510	0.507	0.505	0.509
Mother's age	26.443 (5.595)	24.922 (5.328)	27.159 (5.563)	24.460 (5.136)	24.212 (5.005)	24.631 (5.220)
WIC participation	0.507	0.726	0.399	0.735	0.785	0.692
1st trimester prenatal care	0.711	0.634	0.752	0.633	0.608	0.657
Mother black	0.333	1.000	0.000	0.472	1.000	0.000
Race other	0.019	0.000	0.000	0.014	0.000	0.000
Mother educ.: less than HS	0.210	0.234	0.198	0.305	0.271	0.336
Mother educ.: some college	0.303	0.338	0.286	0.298	0.318	0.281
Mother educ.: college	0.231	0.089	0.300	0.045	0.037	0.050
Prepregnancy smoking	0.145	0.103	0.169	0.209	0.116	0.295
Pregnancy smoking	0.115	0.077	0.136	0.171	0.088	0.249
Prepregnancy diabetes	0.007	0.009	0.006	0.007	0.008	0.006
Prepregnancy hypertension	0.020	0.032	0.014	0.022	0.030	0.015
Previous cesarean	0.166	0.176	0.161	0.164	0.170	0.159
Child first born	0.315	0.287	0.330	0.299	0.289	0.309
Length of gestation (weeks)	38.780 (0.904)	38.698 (0.922)	38.823 (0.892)	38.754 (0.916)	38.701 (0.925)	38.804 (0.904)
Medicaid participation	0.537	0.761	0.426	1	1	1
Observations	408,214	135,914	264,089	219,175	103,444	112,501

*Note:* Standard deviations for continuous variables are in parentheses.

**Table 2:** The effect of GWG and BMI on infant outcomes.

Variables	(1)	(2)	(3)	(4)
	Birth weight	Low birth weight	High birth weight	NICU admission
*All mothers (4,08,214)*				
BMI: obese	74.7870⁣^∗∗∗^	−0.0101⁣^∗∗∗^	0.0258⁣^∗∗∗^	0.0014
	(2.9474)	(0.0015)	(0.0022)	(0.0014)
BMI: overweight	23.6011⁣^∗∗∗^	−0.0044⁣^∗∗∗^	0.0069⁣^∗∗∗^	0.0008
	(2.0589)	(0.0011)	(0.0015)	(0.0010)
BMI: underweight	−29.5534⁣^∗∗∗^	0.0111⁣^∗∗∗^	−0.0015	0.0001
	(3.9724)	(0.0028)	(0.0023)	(0.0020)
Excessive weight gain	57.9948⁣^∗∗∗^	−0.0033⁣^∗∗∗^	0.0234⁣^∗∗∗^	0.0012
	(1.7112)	(0.0008)	(0.0013)	(0.0008)
Inadequate weight gain	−36.1857⁣^∗∗∗^	0.0087⁣^∗∗∗^	−0.0040⁣^∗∗∗^	0.0017⁣^∗^
	(1.7903)	(0.0010)	(0.0011)	(0.0009)
*R*-squared	0.1735	0.0300	0.0246	0.0051

*Black mothers (1,35,914)*				
BMI: obese	64.9388⁣^∗∗∗^	−0.0122⁣^∗∗∗^	0.0130⁣^∗∗∗^	0.0013
	(4.7703)	(0.0030)	(0.0025)	(0.0023)
BMI: overweight	21.2122⁣^∗∗∗^	−0.0057⁣^∗∗^	0.0020	0.0006
	(3.5183)	(0.0023)	(0.0018)	(0.0017)
BMI: underweight	−36.0382⁣^∗∗∗^	0.0266⁣^∗∗∗^	0.0003	0.0056
	(7.1824)	(0.0063)	(0.0028)	(0.0034)
Excessive weight gain	48.7238⁣^∗∗∗^	−0.0057⁣^∗∗∗^	0.0126⁣^∗∗∗^	0.0020
	(2.9909)	(0.0018)	(0.0016)	(0.0014)
Inadequate weight gain	−26.8686⁣^∗∗∗^	0.0090⁣^∗∗∗^	−0.0023⁣^∗^	0.0007
	(2.9483)	(0.0020)	(0.0014)	(0.0014)
*R*-squared	0.1637	0.0420	0.0127	0.0044

*White mothers (2,64,089)*				
BMI: obese	80.3488⁣^∗∗∗^	−0.0075⁣^∗∗∗^	0.0356⁣^∗∗∗^	0.0011
	(3.8320)	(0.0015)	(0.0032)	(0.0018)
BMI: overweight	24.3994⁣^∗∗∗^	−0.0034⁣^∗∗∗^	0.0095⁣^∗∗∗^	0.0006
	(2.5836)	(0.0011)	(0.0021)	(0.0013)
BMI: underweight	−27.3073⁣^∗∗∗^	0.0046	−0.0021	−0.0021
	(4.8899)	(0.0030)	(0.0031)	(0.0025)
Excessive weight gain	63.0625⁣^∗∗∗^	−0.0020⁣^∗∗^	0.0295⁣^∗∗∗^	0.0009
	(2.1178)	(0.0008)	(0.0017)	(0.0010)
Inadequate weight gain	−42.9008⁣^∗∗∗^	0.0080⁣^∗∗∗^	−0.0060⁣^∗∗∗^	0.0029⁣^∗∗^
	(2.3070)	(0.0011)	(0.0017)	(0.0011)
*R*-squared	0.1803	0.0247	0.0322	0.0064

*Note:* All regressions account for child's gender, maternal age dummies, education, first-trimester prenatal care, prepregnancy diabetes and hypertension, prepregnancy and pregnancy smoking, previous caesarian section, first-born status, WIC participation, length of gestation, and year and county fixed effects. Standard errors are clustered on mother's ID.

⁣^∗∗∗^*p* < 0.01.

⁣^∗∗^*p* < 0.05.

⁣^∗^*p*  <  0.1.

**Table 3:** The effect of GWG and BMI on maternal outcomes.

Variables	(1)	(2)	(3)	(4)	(5)
	Breastfeeding	Precipitous labor	Labor augmentation	Induced labor	Cesarean section
*All mothers (4,08,214)*					
BMI: obese	0.0218⁣^∗∗∗^	−0.0049⁣^∗∗∗^	−0.0027	0.0245⁣^∗∗∗^	0.0192⁣^∗∗∗^
	(0.0034)	(0.0018)	(0.0037)	(0.0039)	(0.0022)
BMI: overweight	0.0104⁣^∗∗∗^	−0.0015	−0.0050⁣^∗^	0.0121⁣^∗∗∗^	0.0052⁣^∗∗∗^
	(0.0024)	(0.0014)	(0.0027)	(0.0028)	(0.0015)
BMI: underweight	0.0055	−0.0010	−0.0013	−0.0177⁣^∗∗∗^	0.0023
	(0.0048)	(0.0028)	(0.0056)	(0.0056)	(0.0027)
Excessive weight gain	0.0060⁣^∗∗∗^	−0.0017	0.0046⁣^∗∗^	0.0147⁣^∗∗∗^	0.0053⁣^∗∗∗^
	(0.0019)	(0.0011)	(0.0022)	(0.0023)	(0.0012)
Inadequate weight gain	−0.0058⁣^∗∗∗^	0.0038⁣^∗∗∗^	−0.0043⁣^∗^	−0.0126⁣^∗∗∗^	−0.0042⁣^∗∗∗^
	(0.0021)	(0.0012)	(0.0024)	(0.0025)	(0.0013)
*R*-squared	0.0162	0.0234	0.0461	0.0685	0.0417

*Black mothers (1,35,914)*					
BMI: obese	0.0326⁣^∗∗∗^	−0.0050	0.0024	0.0379⁣^∗∗∗^	0.0133⁣^∗∗∗^
	(0.0061)	(0.0032)	(0.0061)	(0.0064)	(0.0036)
BMI: overweight	0.0180⁣^∗∗∗^	0.0001	−0.0041	0.0197⁣^∗∗∗^	−0.0004
	(0.0046)	(0.0024)	(0.0047)	(0.0049)	(0.0026)
BMI: underweight	0.0002	−0.0037	−0.0213⁣^∗∗^	−0.0146	0.0089⁣^∗^
	(0.0101)	(0.0055)	(0.0105)	(0.0106)	(0.0051)
Excessive weight gain	0.0056	−0.0011	0.0013	0.0072⁣^∗^	0.0073⁣^∗∗∗^
	(0.0038)	(0.0020)	(0.0039)	(0.0041)	(0.0022)
Inadequate weight gain	−0.0061	0.0049⁣^∗∗^	−0.0070⁣^∗^	−0.0159⁣^∗∗∗^	−0.0052⁣^∗∗^
	(0.0038)	(0.0021)	(0.0039)	(0.0041)	(0.0021)
*R*-squared	0.0274	0.0155	0.0522	0.0709	0.0472

*White mothers (2,64,089)*					
BMI: obese	0.0114⁣^∗∗∗^	−0.0038⁣^∗^	−0.0038	0.0184⁣^∗∗∗^	0.0217⁣^∗∗∗^
	(0.0041)	(0.0023)	(0.0047)	(0.0051)	(0.0027)
BMI: overweight	0.0067⁣^∗∗^	−0.0026	−0.0045	0.0092⁣^∗∗∗^	0.0078⁣^∗∗∗^
	(0.0028)	(0.0016)	(0.0034)	(0.0036)	(0.0018)
BMI: underweight	0.0071	0.0003	0.0080	−0.0210⁣^∗∗∗^	−0.0011
	(0.0055)	(0.0033)	(0.0067)	(0.0068)	(0.0033)
Excessive weight gain	0.0055⁣^∗∗^	−0.0020	0.0072⁣^∗∗∗^	0.0184⁣^∗∗∗^	0.0039⁣^∗∗∗^
	(0.0022)	(0.0013)	(0.0027)	(0.0029)	(0.0015)
Inadequate weight gain	−0.0049⁣^∗^	0.0031⁣^∗∗^	−0.0038	−0.0117⁣^∗∗∗^	−0.0026
	(0.0025)	(0.0015)	(0.0030)	(0.0032)	(0.0016)
*R*-squared	0.0121	0.0305	0.0439	0.0693	0.0405

*Note:* All regressions account for child's gender, maternal age dummies, education, first trimester prenatal care, prepregnancy diabetes and hypertension, prepregnancy and pregnancy smoking, previous caesarian section, first-born status, WIC participation, length of gestation, and year and county fixed effects. Standard errors are clustered on mother's ID.

⁣^∗∗∗^*p* < 0.01.

⁣^∗∗^*p* < 0.05.

⁣^∗^*p*  <  0.1.

**Table 4:** The effect of GWG and BMI on infant outcomes among Medicaid mothers.

Variables	(1)	(2)	(3)	(4)
	Birth weight	Low birth weight	High birth weight	NICU admission
*Medicaid mothers (2,19,175)*				
BMI: obese	81.1353⁣^∗∗∗^	−0.0134⁣^∗∗∗^	0.0207⁣^∗∗∗^	0.0009
	(4.1357)	(0.0023)	(0.0027)	(0.0020)
BMI: overweight	27.2841⁣^∗∗∗^	−0.0056⁣^∗∗∗^	0.0040⁣^∗∗^	0.0001
	(3.0031)	(0.0018)	(0.0018)	(0.0014)
BMI: underweight	−30.4018⁣^∗∗∗^	0.0109⁣^∗∗^	0.0014	−0.0002
	(5.4531)	(0.0043)	(0.0027)	(0.0027)
Excessive weight gain	57.4104⁣^∗∗∗^	−0.0037⁣^∗∗∗^	0.0180⁣^∗∗∗^	0.0039⁣^∗∗∗^
	(2.5496)	(0.0014)	(0.0016)	(0.0012)
Inadequate weight gain	−33.7303⁣^∗∗∗^	0.0110⁣^∗∗∗^	−0.0016	0.0027⁣^∗∗^
	(2.5903)	(0.0017)	(0.0014)	(0.0013)
*R*-squared	0.1674	0.0370	0.0169	0.0064

*Black Medicaid mothers (1,03,444)*				
BMI: obese	64.9485⁣^∗∗∗^	−0.0122⁣^∗∗∗^	0.0124⁣^∗∗∗^	0.0024
	(5.7636)	(0.0037)	(0.0029)	(0.0027)
BMI: overweight	24.5832⁣^∗∗∗^	−0.0064⁣^∗∗^	0.0028	0.0005
	(4.2738)	(0.0028)	(0.0020)	(0.0020)
BMI: underweight	−35.1205⁣^∗∗∗^	0.0243⁣^∗∗∗^	0.0027	0.0028
	(8.4282)	(0.0077)	(0.0032)	(0.0039)
Excessive weight gain	46.4023⁣^∗∗∗^	−0.0057⁣^∗∗^	0.0094⁣^∗∗∗^	0.0031⁣^∗^
	(3.6219)	(0.0023)	(0.0019)	(0.0017)
Inadequate weight gain	−25.9391⁣^∗∗∗^	0.0092⁣^∗∗∗^	−0.0024	0.0017
	(3.5368)	(0.0025)	(0.0016)	(0.0017)
*R*-squared	0.1615	0.0423	0.0119	0.0065

*White Medicaid mothers (1,12,501)*				
BMI: obese	95.5639⁣^∗∗∗^	−0.0131⁣^∗∗∗^	0.0311⁣^∗∗∗^	−0.0004
	(6.0445)	(0.0027)	(0.0046)	(0.0029)
BMI: overweight	28.6285⁣^∗∗∗^	−0.0043⁣^∗∗^	0.0053⁣^∗^	−0.0002
	(4.2953)	(0.0022)	(0.0031)	(0.0021)
BMI: underweight	−28.7441⁣^∗∗∗^	0.0041	0.0015	−0.0022
	(7.2542)	(0.0050)	(0.0040)	(0.0037)
Excessive weight gain	67.3303⁣^∗∗∗^	−0.0018	0.0256⁣^∗∗∗^	0.0049⁣^∗∗∗^
	(3.6374)	(0.0017)	(0.0026)	(0.0017)
Inadequate weight gain	−43.3389⁣^∗∗∗^	0.0120⁣^∗∗∗^	−0.0021	0.0040⁣^∗∗^
	(3.8702)	(0.0022)	(0.0025)	(0.0019)
*R*-squared	0.1766	0.0350	0.0251	0.0088

*Note:* All regressions account for child's gender, maternal age dummies, education, first trimester prenatal care, prepregnancy diabetes and hypertension, prepregnancy and pregnancy smoking, previous caesarian section, first-born status, WIC participation, length of gestation, and year and county fixed effects. Standard errors are clustered on mother's ID.

⁣^∗∗∗^*p* < 0.01.

⁣^∗∗^*p* < 0.05.

⁣^∗^*p*  <  0.1.

**Table 5:** The effect of GWG and BMI on maternal outcomes among Medicaid mothers.

Variables	(1)	(2)	(3)	(4)	(5)
	Breastfeeding	Precipitous labor	Labor augmentation	Induced labor	Cesarean section
*Medicaid mothers (2,19,175)*					
BMI: obese	0.0302⁣^∗∗∗^	−0.0019	−0.0055	0.0352⁣^∗∗∗^	0.0099⁣^∗∗∗^
	(0.0051)	(0.0027)	(0.0052)	(0.0056)	(0.0030)
BMI: overweight	0.0140⁣^∗∗∗^	0.0021	−0.0052	0.0213⁣^∗∗∗^	−0.0003
	(0.0038)	(0.0020)	(0.0040)	(0.0042)	(0.0021)
BMI: underweight	0.0024	−0.0024	−0.0064	−0.0146⁣^∗^	0.0007
	(0.0072)	(0.0039)	(0.0075)	(0.0078)	(0.0037)
Excessive weight gain	0.0078⁣^∗∗^	−0.0027	−0.0002	0.0174⁣^∗∗∗^	0.0056⁣^∗∗∗^
	(0.0032)	(0.0017)	(0.0032)	(0.0035)	(0.0018)
Inadequate weight gain	−0.0101⁣^∗∗∗^	0.0032⁣^∗^	−0.0012	−0.0140⁣^∗∗∗^	−0.0033⁣^∗^
	(0.0033)	(0.0018)	(0.0034)	(0.0036)	(0.0018)
*R*-squared	0.0179	0.0201	0.0470	0.0686	0.0460

*Black Medicaid mothers (1,03,444)*					
BMI: obese	0.0370⁣^∗∗∗^	−0.0045	0.0021	0.0365⁣^∗∗∗^	0.0102⁣^∗∗^
	(0.0074)	(0.0038)	(0.0074)	(0.0077)	(0.0043)
BMI: overweight	0.0217⁣^∗∗∗^	0.0022	−0.0045	0.0221⁣^∗∗∗^	−0.0017
	(0.0056)	(0.0029)	(0.0057)	(0.0059)	(0.0031)
BMI: underweight	0.0033	−0.0038	−0.0222⁣^∗^	−0.0244⁣^∗∗^	0.0064
	(0.0118)	(0.0064)	(0.0123)	(0.0123)	(0.0059)
Excessive weight gain	0.0089⁣^∗^	−0.0020	−0.0010	0.0061	0.0057⁣^∗∗^
	(0.0047)	(0.0025)	(0.0047)	(0.0050)	(0.0027)
Inadequate weight gain	−0.0062	0.0055⁣^∗∗^	−0.0014	−0.0204⁣^∗∗∗^	−0.0052⁣^∗∗^
	(0.0047)	(0.0025)	(0.0047)	(0.0049)	(0.0025)
*R*-squared	0.0251	0.0162	0.0519	0.0680	0.0486

*White Medicaid mothers (1,12,501)*					
BMI: obese	0.0182⁣^∗∗^	0.0009	−0.0096	0.0355⁣^∗∗∗^	0.0094⁣^∗∗^
	(0.0072)	(0.0037)	(0.0074)	(0.0082)	(0.0042)
BMI: overweight	0.0067	0.0013	−0.0038	0.0218⁣^∗∗∗^	0.0007
	(0.0052)	(0.0028)	(0.0055)	(0.0060)	(0.0029)
BMI: underweight	0.0020	−0.0013	0.0040	−0.0103	−0.0033
	(0.0093)	(0.0049)	(0.0096)	(0.0102)	(0.0048)
Excessive weight gain	0.0056	−0.0035	0.0006	0.0289⁣^∗∗∗^	0.0051⁣^∗∗^
	(0.0042)	(0.0022)	(0.0045)	(0.0049)	(0.0025)
Inadequate weight gain	−0.0128⁣^∗∗∗^	0.0005	−0.0030	−0.0069	−0.0008
	(0.0047)	(0.0025)	(0.0049)	(0.0053)	(0.0026)
*R*-squared	0.0153	0.0295	0.0446	0.0732	0.0455

*Note:* All regressions account for child's gender, maternal age dummies, education, first trimester prenatal care, prepregnancy diabetes and hypertension, prepregnancy and pregnancy smoking, previous caesarian section, first-born status, WIC participation, length of gestation, and year and county fixed effects. Standard errors are clustered on mother's ID.

⁣^∗∗∗^*p* < 0.01.

⁣^∗∗^*p* < 0.05.

⁣^∗^*p*  <  0.1.

## Data Availability

Data used in this study are restricted. The data could be obtained from the South Carolina Department of Health and Environmental Control. The authors would be willing to assist potential applicants with the data application process.

## Endnotes

^1^Due to rounding, these numbers add up to greater than 100.

^2^A comprehensive overview of the literature on the effects of GWG and prepregnancy BMI can be found in IOM and NRC [5] by Viswathanan et al. [54].

^3^However, the estimates by Cawley and Meyerhoefer [25] specifically exclude pregnant women.

^4^While NICU costs did not differ between infants of obese and nonobese mothers, since the probability of NICU admission is higher for obese mothers, the total costs of being obese, in terms of NICU admissions, are higher.

^5^ https://www.mayoclinic.org/tests-procedures/labor-induction/about/pac-20385141.

^6^Negrini et al. [55] estimate that the Medicaid cost of a C-section is about $4500 higher than that of a vaginal delivery. There are, additionally, worse neonate and mother outcomes from C-section deliveries that add to the cost.

^7^This ranking is based on information from https://assets.americashealthrankings.org/app/uploads/ahr_2023annual_comprehensivereport_final2-web.pdf.
